# Clinical and Genetic Characteristics of Patients with Essential Tremor Who Develop Parkinson’s Disease

**DOI:** 10.3390/medicina61071184

**Published:** 2025-06-29

**Authors:** Gulseren Buyukserbetci, Hilmi Bolat, Ummu Serpil Sari, Gizem Turan, Ayla Solmaz Avcikurt, Figen Esmeli

**Affiliations:** 1 Neurology Department, Faculty of Medicine, Balikesir University, 10145 Balikesir, Turkey; userpilsari@gmail.com (U.S.S.); gizemturan.1994@gmail.com (G.T.); fesmeli@hotmail.com (F.E.); 2 Medical Genetics Department, Faculty of Medicine, Balikesir University, 10145 Balikesir, Turkey; hilmi_bolat@hotmail.com; 3 Department of Medical Biology, Faculty of Medicine, Balikesir University, 10145 Balikesir, Turkey; aylaavcikurt@gmail.com

**Keywords:** Parkinson’s disease, essential tremor, genetic, variants

## Abstract

*Background and Objectives:* Essential tremor (ET) is a common neurological disorder, typically presenting as bilateral, rhythmic, and symmetric kinetic or postural tremors. In contrast, Parkinson’s disease (PD) is a progressive neurodegenerative disorder, characterized by resting tremor, rigidity, bradykinesia, and postural instability. Although both disorders involve tremor, ET and PD differ in clinical presentation and pathophysiology: ET generally involves action tremor and has a strong familial component, while PD more commonly presents with resting tremor and a weaker family history. A subset of ET patients may develop Parkinsonian features over time, although the relationship between ET and subsequent PD remains unclear. Genetic studies have identified only a few pathogenic variants in ET, suggesting it develops as a result of multifactorial genetic and environmental influences rather than simple Mendelian inheritance. ET is also recognized as a risk factor for developing PD, although the underlying mechanisms remain poorly understood. This study aimed to clarify potential genetic overlaps and distinctions in patients diagnosed with both ET and PD. *Materials and Methods:* We retrospectively analyzed 40 patients with a family history of ET or PD who were initially diagnosed with ET and later developed PD. Genetic screening and clinical assessments were conducted to investigate associated variants and clinical features. *Results:* Among these 40 patients, 17 different mutations were detected in 16 individuals. Three pathogenic or likely pathogenic variants were identified. The clinical characteristics and treatment responses of these patients were reviewed in relation to their genetic findings. Notably, none of the identified variants had previously been reported in association with PD following ET. *Conclusions:* A comprehensive clinical and genetic evaluation of ET patients who develop PD may offer insights into the underlying pathophysiology and inform future therapeutic strategies. Our findings support the need for further studies to explore the genetic landscape of patients with overlapping ET and PD features.

## 1. Introduction

Essential tremor (ET) is a complex neurological disorder that typically presents as bilateral, rhythmic, and symmetric kinetic or postural tremors. It affects approximately 1% of the general population and up to 5% of individuals over the age of 65 [1]. Action tremors frequently impair activities such as writing, drinking, eating, and various other aspects of daily living. Although ET is not a fatal condition, it can have a significant negative impact on both daily functioning and psychological well-being. The disorder primarily manifests as postural or kinetic tremors of the arms and hands but may also involve the head, legs, voice, and other body regions [2].

Parkinson’s disease (PD), like ET, is a common movement disorder in adults, affecting approximately 1% of individuals aged over 65 years. Although ET and PD are considered distinct diseases, both present with tremor as a prominent clinical feature. While the etiology of ET remains unclear, some individuals with ET may develop additional Parkinsonian features, such as rigidity, bradykinesia, and postural instability. However, the relationship between this clinical overlap and disease progression is not yet fully understood [3].

Despite numerous studies investigating the genetic etiology of ET, the hereditary basis remains largely unexplained, owing to factors such as misdiagnosis, genetic heterogeneity, and incomplete penetrance of risk alleles. Familial clustering of ET cases underscores the importance of genetic factors in this disorder. Twin studies have shown concordance rates of 69–93% in monozygotic twins and 27–29% in dizygotic twins, suggesting that both genetic and environmental factors contribute to the phenotype. This finding indicates a complex interplay in which genetics plays a significant role but is modulated by nongenetic influences [4].

Although ET has a strong genetic component, successful genetic analyses have been limited by several confounding factors. These include frequent misdiagnosis, the presence of genetic heterogeneity, incomplete penetrance of risk alleles, and the inability to analyze certain genomic regions, such as introns, long noncoding genes, or short noncoding RNAs, such as microRNAs. In addition, environmental influences further complicate the genetic landscape of ET. Consequently, only a small number of pathogenic variants have been reported. This complexity suggests that ET inheritance deviates from simple Mendelian patterns and involves multifactorial genetic and environmental interactions [5].

Specific candidate genes have been identified through studies analyzing families with multiple ET cases, such as *FUS*, *TENM4*, *STK32B*, *LINGO1*, *SORT1*, *SCN4A*, *NOS3*, *KCNS2*, *HAPLN4/BRAL2*, and *USP46*, with variants often unique to families from diverse ethnic backgrounds [6]. However, past genome-wide association studies (GWASs) aimed at pinpointing ET loci have been limited by small cohort sizes, with no statistically significant associations found for any genomic region [1]. Genetic linkage studies conducted in families with multiple affected members have identified three distinct genomic loci associated with ET: 3q13 (ETM1; OMIM 190300), 2p22-24 (ETM2; OMIM 602134), and 6p23 (ETM3; OMIM 611456). However, despite these linkage signals, causal mutations within these chromosomal regions have not yet been definitively characterized [2]. This highlights the complexity of ET genetics and suggests that additional genetic or regulatory elements within these loci may contribute to disease pathogenesis. In summary, while familial studies have uncovered several candidate genes for ET harboring rare variants, GWASs have revealed multiple risk loci with modest effects, emphasizing the multifactorial and polygenic nature of the genetics of ET and the need for larger, ethnically diverse cohorts to clarify its hereditary mechanisms.

A large genome-wide association study, including 16,480 ET cases and nearly 1.94 million controls, found 12 variants across 11 loci, with 7 highlighted as putative causal genes (e.g., *CA3*, *CPLX1*). The results also suggested shared genetic correlations with Parkinson’s disease, anxiety, and depression [7].

Numerous studies have investigated the associations of PD risk variants in ET patients. Among the most significant genetic links are the genes *LINGO1*, *HTRA2*, and *DNAJC13*, which have also been implicated in dystonia. However, extensive sequencing studies in large PD cohorts have produced conflicting or negative results regarding the associations of *HTRA2* and *DNAJC13* variants with PD risk [4]. For example, a comprehensive analysis of *DNAJC13*, *HTRA2*, and other PD-related genes in thousands of PD patients and controls revealed no statistically significant associations after multiple testing correction. Some nominal associations, such as nonsynonymous variants in *DNAJC13*, are more frequent in controls than in PD patients, undermining their role as risk factors. Despite this, these genes continue to be studied for their potential involvement in PD pathogenesis and related movement disorders, including dystonia and ET, owing to their functional roles in neuronal processes. Therefore, while *LINGO1*, *HTRA2*, and *DNAJC13* remain important candidates linking ET, PD, and dystonia, definitive evidence supporting their direct contribution to PD risk is still lacking and requires further investigation [8].

Through genome-wide association studies (GWASs) of ET, associations with common variants located in intronic regions of *LINGO1* have been found. Specifically, multiple studies have identified the *LINGO1* intronic variant rs9652490 as a risk factor for ET across different populations, although others have yielded conflicting results, suggesting the possibility of allelic heterogeneity and environmental interactions. *LINGO1* encodes a protein that acts as a strong negative regulator of neuronal survival and plays a critical role in the central nervous system by modulating axonal regeneration and oligodendrocyte maturation. The *LINGO1* protein contains leucine-rich repeat and immunoglobulin-like domains that enable it to form complexes with receptors such as the Nogo-66 receptor (NgR1), thereby activating signaling pathways that inhibit oligodendrocyte differentiation and myelination [6].

The *DNAJC13* p.N855S (p.Asn855Ser) mutation, initially identified as a cause of autosomal dominant PD, is known to regulate clathrin dynamics and endosomal trafficking. This mutation has also been detected in two unrelated patients with ET, suggesting a potential overlap in the genetic basis of PD and ET [3].

Whole-exome sequencing and pedigree analysis of a six-generation Turkish family exhibiting both PD and ET phenotypes was used to identify the mitochondrial serine protease variant *HTRA2* p.G399S as the likely causative allele for hereditary ET in these families. Individuals heterozygous for *HTRA2* p.G399S presented ET symptoms typically after the age of 70, whereas homozygous carriers developed ET with earlier onset and more severe postural and kinetic tremors and additionally manifested Parkinsonian features in middle age. This dosage-dependent effect suggests that heterozygosity for the allele predisposes to ET, whereas homozygosity leads to a combined ET–PD phenotype [2].

Although ET is currently recognized as a disorder with a strong genetic component, its genetic basis remains largely elusive. Epidemiological and neuropathological studies demonstrate a close relationship between ET and PD [9,10]. Moreover, it is well established that ET increases the risk of developing PD.

With our study, we aim to contribute to this field by elucidating genetic overlaps and distinctions between ET and PD in patients exhibiting both conditions, thus reinforcing the notion that ET is not strictly monosymptomatic and that genetic factors contributing to ET may also influence susceptibility to PD, reflecting the continuum and complexity of movement disorders.

The primary aim of this study is to explore the genetic overlap between ET and PD, focusing on the identification of genetic variants that may contribute to both conditions. The hypothesis of this research is that specific genetic mutations are shared between ET and PD, influencing their co-occurrence in some individuals. The clinical features of patients with identified mutations and their associations with these genetic variants are discussed in detail.

## 2. Materials and Methods

**Figure 1:**
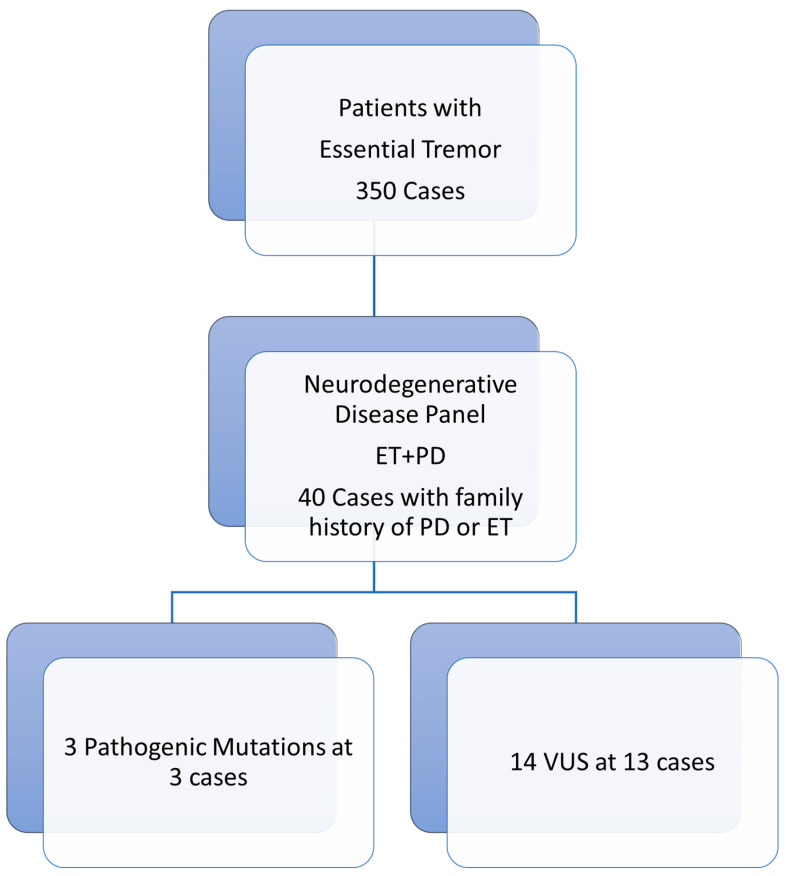
Patient flow diagram: ET: Essential tremor; PD: Parkinson’s disease; VUS: Variant of uncertain significance.

Our study is a retrospective analysis. Between May 2022 and 2024, we screened 350 patients diagnosed with ET who presented to the Movement Disorders Clinic of the Neurology Department at Balikesir University Research Hospital. Of these, 40 who developed PD during follow-up, had first- or second-degree relatives diagnosed with either essential tremor or Parkinson’s disease, and underwent neurodegenerative disease panel testing at the Department of Medical Genetics of Balikesir University were included in the study (Figure 1).

For these patients, information was recorded regarding demographic data, family history characteristics, clinical presentations, durations of ET and PD, neurological examinations, medical treatments used, and additional chronic diseases. Genetic analysis based on a neurodegenerative disease panel revealed various mutations in 16 of the 40 patients (Figure 1).

This methodology allowed us to investigate the genetic underpinnings of patients with ET who subsequently developed PD, focusing on those with familial predisposition and accessible data from comprehensive genetic screening.

The diagnosis of ET in this study was established according to the Consensus Statement of the Movement Disorder Society (MDS) on tremor, which requires the presence of bilateral upper limb action tremors (postural and/or kinetic) of at least three years’ duration, with or without tremors in other locations (such as the head or voice), and the absence of other neurological signs, such as dystonia, ataxia, or Parkinsonism [11].

The diagnosis of PD was made based on the clinical diagnostic criteria of the United Kingdom Parkinson’s Disease Society Brain Bank and the Movement Disorder Society (MDS) criteria, both of which are internationally recognized standards for PD diagnosis.

ET was assessed via the Fahn–Tolosa–Marin Clinical Rating Scale for Tremor (FTM), a widely used and validated tool for quantifying tremor severity and its impact on daily living in ET patients [12]. For PD, clinical evaluation was performed using the Movement Disorder Society Unified Parkinson’s Disease Rating Scale (MDS-UPDRS), and PD staging was determined via the Hoehn–Yahr scale [13,14].

### 2.1. Genetic Testing

#### 2.1.1. DNA Isolation

Informed consent was obtained from the parents and affected and/or normal family members of all patients, and 5–10 cc of peripheral venous blood was collected from patients into EDTA tubes. Genomic DNA (gDNA) was extracted from these blood samples using an ExgeneTM Blood SV isolation kit (GeneAll Biotechnology, Seoul, Republic of Korea) and following the manufacturer’s protocol.

#### 2.1.2. Clinical Exome SEQUENCING (CES)

Exome sequencing libraries for use with a Human Comprehensive Exome Panel (Twist Bioscience, South San Francisco, CA, USA) were prepared according to the manufacturer’s instructions. Following the target enrichment process, the libraries were sequenced on a DNBSEQ-G400 (MGI Tech, Shenzhen, China) at 80–100× on-target depth with 150 bp paired ends. The raw reads were cleaned from adapter contamination during the demultiplexing stage; therefore, no further adapter cleaning was performed on the FASTQ files. Alignment to GRCh38 was performed via BWA-MEM 0.7.17 [15]. Subsequent sorting, duplicate marking, and base score recalibration steps were performed via GATK. Variant calling was performed via the GATK Haplotype Caller, and low-quality variants were eliminated based on strand bias, read depth, call quality, and other related parameters [16].

#### 2.1.3. Variant Analysis

High-quality variants were subjected to functional annotation via the Variant Effect Predictor from ENSEMBL [17], prioritizing for rare variants (MAF < 1%) with high impact, unknown significance, and/or potential splice effects. Other variants with potential effects on the observed phenotype were also analyzed. The pathogenicity of the detected variants was evaluated based on criteria from [18]. Variants of interest were visually checked via the Integrative Genomics Viewer (IGV) and compared against an in-house disease variant database from the Balikesir Genetic Diagnostic Center, Balikesir, Turkey [19].

First, we filtered 300 genes associated with neurodegenerative diseases (Appendix A). Human Phenotype Ontology was then used for phenotypic filtering, and Online Mendelian Inheritance in Man (OMIM, https://www.omim.org/, accessed on 1 March 2024) was used for the gene sets. To assess their novelty, identified variants were checked against The Human Genome Mutation Database (HGMD, http://www.hgmd.cf.ac.uk/ac/index.php, accessed on 1 March 2024), Franklin (https://franklin.genoox.com/clinical-db/home, accessed on 1 March 2024), and VarSome (https://varsome.com/, accessed on 1 March 2024). The pathogenicity score of new variants was interpreted via the silico variant prediction program Mutation Taster, together with annotation-dependent depletion analysis.

Ethical approval for the study was obtained from the Balikesir University Ethics Committee (Ethics Committee approval number: 2024/21, dated 21 February 2024).

## 3. Results

Among the 40 patients in our study, 17 different mutations were detected in 16 individuals. Of the mutation-positive patients, 62.5% were male and 37.5% were female, with a mean age of 62.18 ± 16.22 years. The average duration of PD was 3.75 ± 3.19 years, whereas the mean duration of ET was 13.62 ± 12.31 years. The average interval between ET diagnosis and subsequent PD diagnosis was 10.75 ± 10.40 years. Additionally, 31.25% of these patients had consanguineous family histories. Of the patients with ET, 56.25% presented with tremors in both upper extremities, 31.25% presented with tremors in the right upper extremity, and 12.5% presented with tremors in the left upper extremity.

### Clinical Features of Patients with Pathogenic/Likely Pathogenic Variants

In our study, a pathogenic variant, NM_001199397.3(*NEK1*):c.3191C>G (p.Ser1064Ter), was identified in a 60-year-old male patient (Patient No. 1 in Table 1 and Table 2). The patient has no parental consanguinity; however, his father had ET. Clinically, the patient has a 10-year history of postural and kinetic tremors in the right upper extremity, with the addition of marked bilateral bradykinesia and rigidity in the right upper extremity over the last 5 years. Gait examination revealed a mild anteflexion posture and reduced participation of the right upper extremity during walking. Cognitive function and olfactory test results (anosmia/hyposmia) were normal. The patient’s ET Fahn–Tolosa–Marin Clinical Rating Scale score was 19. Following a PD diagnosis, the Movement Disorder Society-Unified Parkinson’s Disease Rating Scale (MDS-UPDRS) nonmotor symptom score was 2, the motor symptom score was 20, and the Hoehn and Yahr stage was 1. Neurological examination revealed normal cranial nerve function, muscle strength, and sensory assessments, and deep tendon reflexes, with no pathological reflexes observed. Brain MRI revealed no pathology other than mild, diffuse cortical atrophy. The patient was diagnosed with Parkinson’s disease five years after the initial diagnosis of essential tremor.

In our study, a pathogenic *GBA* (NM_000157.4):c.1448T>C (p.Leu483Pro) mutation was identified in our second patient (Patient No. 2 in Table 1 and Table 2); she is 70 years old. The patient, whose parents are consanguineous, has a family history of ET, affecting the mother and two nieces, and is married to a cousin with a diagnosis of PD who is also undergoing follow-up in our clinic. Additionally, both the son and daughter of the patient were diagnosed with limb-girdle myopathy.

This patient has had essential tremor for 30 years and was also diagnosed with Parkinson’s disease two years ago. The neurological examination revealed bilateral postural, action, and resting tremors. Additionally, rigidity in the extremities was more pronounced on the right side than the left, accompanied by hypomimia and bradykinesia. The patient was morbidly obese and also diagnosed with hypertrophic heart failure and type 2 diabetes mellitus.

The patient initially started on propranolol (120 mg) and primidone (250 mg) twice daily for ET symptoms. While a moderate therapeutic effect was initially observed, efficacy diminished over time, accompanied by increased tremor severity. Consequently, onabotulinum toxin A therapy was added to the tremor regimen. The patient exhibited a marked response to the first two injections; however, no benefit was observed with the subsequent two applications, leading to discontinuation of this treatment.

Over the past year, the clinical picture has evolved with the emergence of asymmetric resting tremors and bradykinesia, prompting the initiation of levodopa therapy, which has yielded no significant clinical improvement. The patient did not report any olfactory loss.

On assessment, the Fahn–Tolosa–Marin Clinical Rating Scale score for ET increased from 25 at the initial evaluation to 38 following the diagnosis of PD. The MDS-UPDRS nonmotor symptoms score was 10, the motor symptoms score was 60, and the Hoehn and Yahr (H&Y) stage score was 1. Glove-and-stocking hypoesthesia and hyporeflexia of deep tendon reflexes were observed on neurological examination, while motor examination revealed no muscle weakness. No pathological reflexes were detected.

Nerve conduction studies revealed sensory polyneuropathy. Brain MRI demonstrated chronic ischemic gliotic lesions consistent with Fazekas grade I, as well as mild diffuse cortical atrophy.

A 66-year-old male patient, described as Patient No. 3 in Table 1 and Table 2, has a 20-year history of ET and been followed for PD development over the last 5 years. The patient has no parental consanguinity and no family history of cerebellar system disorders. His family history includes PD in his mother and ET in his father and paternal grandfather. No medical treatment was initially recommended since his ET symptoms were mild. During follow-up, bilateral mild postural and action tremors appeared, accompanied predominantly on the right side by resting tremors, rigidity, and bradykinesia. The remainder of the neurological examination of the patient, who has a medical history of depression, diabetes mellitus, and hyperlipidemia, was normal. Brain MRI revealed mild cortical atrophy. Treatment was initiated with rasagiline, pramipexole, and later levodopa. At evaluation, the Hoehn and Yahr stage was 3, the MDS-UPDRS scores were 10 for nonmotor symptoms and 49 for motor symptoms, and the Fahn–Tolosa–Marin Clinical Tremor Rating Scale score was 10. The patient had a moderate to poor response to treatment.

## 4. Discussion

Parkinson’s disease and essential tremor are prevalent age-related conditions whose clinical manifestations worsen over time, eventually impacting on patients’ quality of life [20,21]. In recent years, advances in genetics have offered new hope for elucidating the pathogenesis of diseases that remain poorly understood. However, the genetic characteristics of ET, a disorder characterized by high genetic and clinical heterogeneity, are still not fully understood. Emerging studies suggest that ET may serve as a risk factor for other neurodegenerative diseases, such as Alzheimer’s and Parkinson’s [22].

Genetic evaluation in ET patients with a strong family history is important for assessing susceptibility to PD and for identifying at-risk individuals. The literature indicates that ET is an independent risk factor for PD, with a significantly higher incidence of PD observed in individuals with ET than in control individuals [23,24]. Furthermore, familial aggregation of ET and PD is frequently reported, and autosomal dominant inheritance patterns in familial ET cases may contribute to an increased risk of developing PD [24].

Therefore, conducting genetic analyses in patients with a family history of ET can be clinically valuable for determining the risk of PD development and for guiding patient monitoring strategies. This approach may enhance understanding regarding the shared genetic basis of ET and PD. Thus, early ET diagnosis may be useful in detecting PD.

We identified three pathogenic/likely pathogenic variants in three patients (Table 1). In our study, one pathogenic variant, NM_001199397.3(*NEK1*):c.3191C>G (p.Ser1064Ter), was identified in a 60-year-old male patient (Patient No. 1 in Table 1 and Table 2). This variant has been previously associated with conditions such as short rib thoracic dysplasia type 6 (with or without polydactyly), orofaciodigital syndrome II, and amyotrophic lateral sclerosis (ALS). The patient has a 10-year history of ET, with the addition of PD over the last 5 years.

Serine/threonine NIMA-related kinases (NEKs), belonging to the kinase family, play key roles in the regulation of the cell cycle. NEK1 is primarily localized in cytoplasm, although a portion of the protein is also found in mitochondria [25]. NEK family members play crucial roles in DNA damage response (DDR) signaling, with some NEKs acting as checkpoint targets inhibited by DNA damage. *NEK1* regulates cell cycle progression, particularly during meiosis, and is involved in gametogenesis and spermatogenesis. It contributes to the assembly of the meiotic spindle and participates in multiple DDR signaling pathways through direct interactions with various DNA repair proteins. Research has linked *NEK1* to several human diseases, including polycystic kidney disease, amyotrophic lateral sclerosis (ALS), glioma, Wilms tumor, thyroid cancer, and prostate cancer. This highlights the multifaceted role of *NEK1* in maintaining genomic integrity and its implications in diverse pathological conditions [26]. At the molecular level, *NEK1* has been shown to stabilize the complex formed between ATR (ATM and Rad3-related) and ATRIP (ATR-interacting protein) [27]. Studies have demonstrated that *NEK1* knockout (KO) cells exhibit an increased tendency toward apoptosis, indicating the mechanistic contribution of *NEK1* deficiency to apoptotic susceptibility [26,28]. Disruptions in cell survival and normal proliferation occur when *NEK1* is mutated or its activity is insufficient [29]. Studies on amyotrophic lateral sclerosis (ALS) patients have linked the *NEK1* gene to multiple cellular functions, including ciliogenesis, the DNA damage response (DDR), microtubule stability, neuronal morphology, and axonal polarity [30]. In another study involving ALS patients, impairments in hippocampal/temporal lobe functions that are not specific to ALS were observed, such as nonverbal memory. Cranial MRI revealed significant atrophy in the hippocampus, along with some degree of atrophy in the caudate nucleus and thalamus, without extension to other brain regions [31]. Tremor may be associated not only with cellular activity in the motor cortex and basal ganglia nuclei but also with increased cellular activity in the ventral intermediate nucleus (VIM) of the thalamus, which serves as a cerebellar relay nucleus [32].

The association of this variant with ET and Parkinsonian features in our patient suggests a possible broader neurological impact of *NEK1* mutations beyond ALS. The absence of cognitive impairment and anosmia, alongside mild Parkinsonism, may indicate early-stage or atypical neurodegenerative involvement linked to this genetic variant.

In our study, a pathogenic *GBA* (NM_000157.4):c.1448T>C (p.Leu483Pro) mutation was identified in our second patient (Patient No. 2 in Table 1 and Table 2). She has had ET for 30 years and was also diagnosed with PD two years ago.

The association between *GBA1* mutations and sporadic PD was first described in 2009. The *GBA* gene is located on chromosome 1q21 and consists of 11 exons. It encodes the lysosomal enzyme β-glucocerebrosidase, which is involved in the hydrolysis of glucosylceramide. While homozygous or compound heterozygous mutations in *GBA1* cause Gaucher’s disease, heterozygous *GBA1* variants have been identified as the most common genetic risk factor for PD, significantly increasing the risk of developing this disease [33,34]. These variants are associated with earlier disease onset, more severe motor symptoms, and accelerated cognitive decline compared with idiopathic PD [34]. Various mechanisms have been proposed to be involved in the pathogenesis of PD, including loss-of-function and toxic gain-of-function processes [35,36]. *GBA1* mutations cause the accumulation of α-synuclein. The aggregation of α-synuclein, along with lysosomal dysfunction, endoplasmic reticulum (ER) stress, neuroinflammation, and the interaction between GCase and the LRRK2 protein, are recognized as significant contributing factors in the pathogenesis of PD. These molecular disturbances impair cellular protein degradation pathways and promote neurodegeneration, thereby increasing susceptibility to PD. Additionally, GCase–LRRK2 interactions further influence lysosomal homeostasis and inflammatory responses, underscoring the multifactorial mechanisms by which *GBA1* mutations increase PD risk [34]. Studies have shown that inhibiting the function of the *GBA1* gene increases α-synuclein release and concurrently elevates the levels of phosphorylated and insoluble α-synuclein in dopaminergic cells [37]. Additionally, GBA-associated Parkinson’s disease (GBA1-PD) brains exhibit Lewy body pathology similar to that of sporadic PD but are reportedly more widespread [38].

In another study, individuals carrying *GBA1* mutations were found to exhibit greater cortical degeneration in the temporal, parietal, and occipital regions than healthy controls and noncarriers [39]. Compared with idiopathic PD patients, patients with GBA1-PD exhibit more severe motor disability and more rapidly progress to Hoehn and Yahr stage 3 disease. Of 50 Ashkenazi Jewish PD patients screened for four homozygous variants, one presented with asymmetric resting tremor at age 48, followed by asymmetric postural tremor eight years later. Another patient developed Parkinsonian symptoms, including resting tremor, starting at age 64. In these patients, tremor was not the dominant symptom, and their responses to L-DOPA treatment ranged from good to moderate [40]. In a Chinese cohort study of PD patients with 301 *GBA1* and 95 *L483P-PD* mutations, 45 (14.86%) patients with GBA1-PD and 16 (16.84%) with L483P-PD presented tremor-dominant symptoms. Compared with noncarrier PD patients, tremor scores (UPDRS tremors) were significantly different, with the GBA1 group having higher scores. However, the study did not detail the tremor types and onset [41]. In contrast to these patients, ours is distinguished by an initial presentation of asymmetric ET that progressed to develop asymmetric resting tremor, a tremor-dominant clinical phenotype, and poor response to medical treatments. This clinical course, characterized by the transition from ET to Parkinsonian resting tremor with limited pharmacological responsiveness, separates our patient from typical ET or tremor-dominant PD cases reported in the literature. However, the clinical course is consistent with the literature on GBA1-PD, characterized by resistance to antiparkinsonian treatments and rapid progression.

In our study, a likely pathogenic variant, NM_001199397.3(*NEK1*):c.3191C>G (p.Ser1064Ter), was identified *TGM6* (NM_198994.3) in a 66-year-old male patient with a 20-year history of ET that has been followed for PD development over the last 5 years (Patient No. 3 in Table 1 and Table 2).

In this patient, the clinical progression from longstanding ET to asymmetric Parkinsonian features with moderate-to-poor response to dopaminergic therapy aligns with the phenotype described in *TGM6* mutation carriers. The absence of cerebellar signs, despite having the mutation and a family history of both ET and PD, further supports the involvement of *TGM6* variants in Parkinsonian syndromes with variable presentations.

*TGM6* encodes transglutaminase 6 (TG6), a member of the transglutaminase enzyme family. Transglutaminases catalyze posttranslational modifications of proteins, specifically the covalent crosslinking of proteins through transamidation reactions. In proteins, this process generally involves the formation of intra- or intermolecular isopeptide bonds between the γ-carboxamide group of a glutamine residue and the ε-amino group of a lysine residue [42]. TG6 is abundantly expressed in the brain, especially in the septal region, basal ganglia, and cerebellum. For example, TG2 can catalyze the formation of α-synuclein crosslinks, which is considered an early step in PD pathogenesis [43]. Heterozygous mutations in the *TGM6* gene located on chromosome 20p13 have been reported to cause autosomal dominant spinocerebellar ataxia type 35 (SCA35). SCA 35 is an autosomal dominant adult-onset neurologic disorder characterized by difficulty walking due to cerebellar ataxia. The age at onset ranges from teenage years to late adulthood, and the disorder is slowly progressing. Additional features may include hand tremors, dysarthria, hyperreflexia, and saccadic eye movements [44]. Our patient was not diagnosed with spinocerebellar ataxia type 35 (SCA35) because of the absence of cerebellar or pyramidal signs and the predominance of Parkinsonism at the clinical presentation. Consistent with the literature, Kui Chen et al. demonstrated that variants in the *TGM6* gene, which encodes transglutaminase 6, can be associated with PD. Their study revealed that *TGM6* variant carriers present typical Parkinsonian features but tend to have slower disease progression. Functional analyses revealed that mutant *TGM6* proteins have reduced enzymatic activity, leading to impaired autophagy and increased α-synuclein accumulation, mechanisms implicated in PD pathogenesis. These findings suggest that *TGM6* may play a role in the pathogenesis of PD through mechanisms involving α-synuclein accumulation and autophagy impairment [45].

There are no reports in the literature of PD developing after ET when associated with *TGM6* variants. Given that the pathogenesis of ET has not been fully elucidated but is thought to involve the cerebellum and its connections, it is possible that the *TGM6* mutation identified in our patient contributes to ET through effects on the cerebellar system. This hypothesis aligns with the known abundant expression of TG6 in cerebellar regions and its involvement in neurodegenerative processes. While *TGM6* variants have been linked to PD and spinocerebellar ataxia type 35, their role in ET or ET preceding PD has not yet been documented.

Although we identified 14 variants of uncertain significance (VUSs) in 13 patients, as shown in Table 1, they are indicated as having uncertain clinical significance owing to the inability to perform family screening (because of the study being retrospective) despite the presence of notable family history characteristics. However, definitive variant classification could be achieved if family genetic testing was possible or if advanced functional studies related to these variants could be conducted.

The genes highlighted in bold in Table 1 were identified as novel variants and are not registered in ClinVar. Even among the variants that are listed in ClinVar, clinical correlation and evidence remain insufficient. This reflects the current challenge in interpreting the pathogenicity and clinical significance of newly discovered or rare genetic variants, especially those classified as VUSs. Without comprehensive family segregation studies or functional analyses, the definitive classification and clinical interpretation of these variants remain limited.

In Patient 14, a VUS was detected in the *GBA* gene, as shown in Table 1 and Table 2. Although, as mentioned above, this mutation was considered potentially explanatory for the patient’s clinical presentation, its clinical significance remains uncertain because family screening could not be performed. The lack of segregation analysis limits definitive interpretation; therefore, the variant is reported to be of uncertain clinical relevance. If family genetic testing or advanced functional studies on this variant were conducted, a clearer classification regarding its pathogenicity could be established.

In our study, 17 variants were identified across 17 genes in 16 cases, including 14 missense mutations, one nonsense mutation, and one splice-site variant. Among these, we detected six novel variants that have not been previously reported. Seven of the variants were absent from both the gnomAD and ClinVar databases. Additionally, three variants found in three separate cases were classified as pathogenic or likely pathogenic and are considered potentially related to the clinical phenotype.

This highlights the genetic heterogeneity and the presence of novel, uncharacterized variants in the cohort. The identification of novel variants not documented in public databases underscores the importance of further functional studies and family segregation analyses to clarify their clinical significance. Moreover, the detection of pathogenic or likely pathogenic variants in clinically relevant genes supports the contribution of genetic factors to disease manifestation in these patients.

The genes listed with VUSs in Table 1 are *NOTCH3*, *FIG4*, *PSEN1*, *TOR1A*, *CCDC88C*, *LRRK2*, *SGCE*, *GRN*, *COL4A1*, *SPAST*, *GBA1*, *DNAJC13*, and *ADH1C.*

Family segregation studies and advanced functional assays remain crucial to resolving VUS classification. Until such data are available, VUS should not be used as the sole basis for clinical decision-making but rather as a prompt for further investigation. The identification of VUS in genes relevant to neurodegenerative diseases highlights the ongoing need for comprehensive genetic and functional research to improve diagnostic accuracy and patient care.

In our cohort of 40 patients, we identified three pathogenic or likely pathogenic variants potentially related to the clinical phenotype in three patients, whereas in the remaining 13 patients, 14 different VUS variants were detected. Since this study is retrospective and family histories were considered, the clinical significance of these variants may change upon re-evaluation. Following progress from clinical and functional studies on these genes, their relevance will become clearer.

A review of the literature revealed that some VUSs have been implicated in other neurological disorders, such as frontotemporal dementia and amyotrophic lateral sclerosis. Although their association with PD and/or ET has not yet been established, such links may be elucidated in future studies.

This underscores the importance of ongoing genetic, clinical, and functional research to better understand the pathogenic potential of VUSs and their possible roles in neurodegenerative diseases, including PD and ET.

Despite therapeutic advances aimed at symptomatic control, no proven treatment currently exists that alters the course or prevents the occurrence of either Parkinson’s disease or essential tremor. Understanding the genetic basis of disease may provide us with the knowledge and tools to design future therapeutic strategies targeting the very molecular events leading to neuronal loss/dysfunction.

## 5. Conclusions

In our study, we identified three pathogenic or likely pathogenic genetic variants in patients who developed PD following ET. We aim to share the clinical features and treatment responses of patients carrying these variants and contribute to the growing body of genetic research. Based on the most current databases and available literature, we conclude that the pathogenic/likely pathogenic variants we identified have not been previously reported in association with the development of Parkinson’s disease following essential tremor. These findings are potentially significant, although further investigation and validation in additional cases are necessary to confirm their relevance.

A limitation of our study is its retrospective design, which prevented the genetic screening of family members. However, family screening is planned for future follow-up.

Most detected mutations were classified as VUSs. All mutation-positive patients continue to be followed clinically at our center.

Although genetic testing has not yet been integrated into routine clinical practice for essential tremor (ET), elucidating its genetic underpinnings remains a critical area of investigation, with the potential to substantially advance our understanding of its pathophysiology. Further studies are needed to identify and validate genes responsible for ET.

Comprehensive clinical and genetic evaluations of ET patients who develop PD may provide valuable insights into disease pathophysiology and guide future therapeutic strategies.

## Figures and Tables

**Table 1:** All patients are heterozygous for the identified variants. ACMG (American College of Medical Genetics and Genomics) Classification: The 28 criteria can be classified into two categories: 16 pathogenic criteria and 12 benign criteria. Depending on the weight of the evidence, the pathogenic criteria are further divided into very strong (PVS1), strong (PS1–4), moderate (PM1–6), and supporting (PP1–5); the benign criteria are divided into standalone (BA1), strong (BS1–4), and supporting (BP1–7). OMIM: Online Mendelian Inheritance in Man Database.

Case	Gene	Exon/Intron	Variant Type	Nucleotide Change	Amino Acid Change	gnomAD v4.1.0 Allele Frequency	ClinVar	ACMG Classification	OMIM Phenotypes
1	*NEK1 (NM_001199397.3)*	31	Nonsense	c.3107C>G	p.Ser1036Ter	0.0002520	Pathogenic	Pathogenic (PVS1, PP5, PM2)	Amyotrophic lateral sclerosis, susceptibility to 24-AD
2	*GBA (NM_000157.4*	10	Missense	c.1448T>C	p.Leu483Pro	-	-	Likely Pathogenic (PS3,PP2,PP5)	Parkinson disease, late-onset, susceptibility to 24-AD
3	*TGM6* *(NM_198994.3)*	Intron 6	Splice-site	c.851-2A>C	-	0.000007435	VUS	Likely pathogenic (PVS1, BS2)	Spinocerebellar ataxia 35-AD
4	*NOTCH3 (NM_000435.3)*	9	Missense	c.1385C>T	p.Thr462Ile	0.000006577	VUS	VUS (PM2)	Cerebral arteriopathy with subcortical infarcts and leukoencephalopathy 1- AD
	*FIG4* *(NM_014845.6)*	7	Missense	c.670C>T	p.Pro224Ser	0.000002489	VUS	VUS (PM2)	Amyotrophic lateral sclerosis 11-AD
5	*PSEN1* *(NM_000021.4)*	7	Missense	c.566A>G	p.Tyr189Cys	0.00006225	VUS	VUS (BS2, PP3, PP2)	Alzheimer disease, type 3, with or without spastic paraparesis-AD, Dementia, frontotemporal-AD, Pick disease-AD
6	*TOR1A* *(NM_000113.3)*	3	Missense	c.467G>A	p.Arg156Gln	0.000003718	VUS	VUS (PM2, BP1)	Dystonia-1, torsion
7	*CCDC88C* *(NM_001080414.4)*	15	Missense	c.2554G>C	p.Asp852His	0.00001611	VUS	VUS (PM2, BP1)	Spinocerebellar ataxia 40-AD
8	*LRRK2* *(NM_198578.4)*	5	Missense	c.470G>T	p.Ser157Ile	-	-	VUS (BP4, PM2)	Parkinson disease 8-AD
9	*NOTCH3* *(NM_000435.3)*	14	Missense	c.2264G>A	p.Gly755Asp	-	-	VUS (PM2, BP4)	Cerebral arteriopathy with subcortical infarcts and leukoencephalopathy 1- AD
10	*SGCE* *(NM_003919.3)*	7	Missense	c.907C>A	p.Pro303Thr	-	-	VUS (PM2, BP1, PP3)	Dystonia-11, myoclonic-AD
11	*GRN (NM_002087.4*	5	Missense	c.430G>A	p.Asp144Asn	0.00001239	VUS	VUS (PM2, PP5, BP1, BP3)	Frontotemporal dementia 2-AD-AR
12	*COL4A1 (NM_001845.6)*	52	Missense	c.4966C>A	p.Arg1656Ser	0.00002726	VUS	VUS (PP2)	Microangiopathy and leukoencephalopathy, pontine, autosomal dominant-AD
13	*SPAST (NM_014946.4)*	6	Missense	c.925C>A	p.Arg309Ser	-	-	VUS (PM1, PM2, PM5, BP4)	Spastic paraplegia 4, autosomal dominant-AD
14	*GBA1 (NM_001005741.3)*	9	Missense	c.1103G>A	p.Arg368His	0.00004213	VUS	VUS (PM1, PM2, BS3, BS4)	Parkinson disease, late-onset, susceptibility to-AD-Mu
15	*DNAJC13 (NM_015268.4)*	40	Missense	c.4544C>G	p.Pro1515Arg	-	-	VUS (PP3, PM2, BP1)	Parkinson disease 21
16	*ADH1C* *(NM_000669.5)*	5	Missense	c.539G>C	p.Gly180Ala	-	-	VUS (PM2, BP1)	Parkinson disease, susceptibility to-AD-Mu

**Table 2:** Patients’ demographic and clinical characteristics.

	AGE	Gender	Duration of PD (Year)	Duration of ET (Year)	Consanguineous Marriage	Family History	Tremor of ET	Examination Findings of Parkinsonism	Medical Treatment	HOEHN JAHR Scale	Fahn–Tolosa–Marin Tremor Rating Scale
1	60	M	6	10	-	Father has ET	Right	Resting tremor Rigidity Bradykinesia/Hypokinesia	Propranolol Rasagiline Pramipexole	1	19
2	70	F	2	30	+	Mother and cousin have ET, another cousin has PD, son and daughter have myopathy.	Right	Resting tremor Rigidity Bradykinesia/Hypokinesia Postural Instability	Propranolol Primidone L-DOPA	1	38
3	66	M	5	20	-	Mother has PD, father and grandfather have ET	Bilateral	Resting tremor Rigidity Bradykinesia/Hypokinesia	Rasagiline Pramipexole L-DOPA	2	40
4	84	M	10	50	-	Father, aunt-and sister and brother have ET	Bilateral	Resting tremor Rigidity Bradykinesia/Hypokinesia	Primidone Pramipexole L-DOPA	1	38
5	54	F	1	3	-	Father has PD	Bilateral	Resting tremor Rigidity	Propranolol Pramipexole	1	36
6	70	M	6	8	-	Mother, grandmother and sister have ET	Right	Resting tremor Rigidity Bradykinesia/Hypokinesia	Rasagiline Pramipexole L-DOPA	2	48
7	43	F	1	6	-	Father has ET.	Right	Resting tremor Rigidity Bradykinesia/Hypokinesia	Rasagiline Pramipexole	1	36
8	20	M	1	11	-	Grandfather has ET	Right	Resting tremor Rigidity Bradykinesia/Hypokinesia	Propranolol	1	42
9	85	M	1	15	-	Father has ET	Left	Resting tremor Rigidity Bradykinesia/Hypokinesia	Primidone Ropinirole	1	42
10	65	M	3	10	-	Father has ET	Bilateral	Resting tremor Rigidity Bradykinesia/Hypokinesia	Propranolol Rasagiline Pramipexole	1	24
11	83	M	1	15	+	Brother has ET	Bilateral	Resting tremor Rigidity Bradykinesia/Hypokinesia	Pramipexole	1	48
12	54	F	1	2	+	Aunt has ET	Bilateral	Resting tremor Rigidity Bradykinesia/Hypokinesia	Propranolol Pramipexole	1	52
13	67	F	4	10	-	Father has ET	Bilateral	Resting tremor Rigidity Bradykinesia/Hypokinesia	Propranolol Pramipexole L-DOPA	2	48
14	61	F	9	22	-	Brother and sister have PD	Left	Resting tremor Rigidity Bradykinesia/Hypokinesia	Pramipexole Propranolol	1	48
15	57	M	8	10	+	Sister has epilepsy and ET	Bilateral	Resting tremor Bradykinesia/Hypokinesia	Rasagiline Pramipexole L-DOPA	1	44
16	56	M	1	10	-	Mother and grandfather have ET	Bilateral	Rigidity Bradykinesia/Hypokinesia	Primidone Rasagiline Pramipexole	1	54

## Data Availability

Data are unavailable due to privacy or ethical restrictions.

## Appendix A

Firstly, We filtered 300 genes (*AARS1*, *AARS2*, *ABCB7*, *ABCD1*, *ABHD12*, *ACOX1*, *ADAR*, *ADCY5*, *ADH1C*, *AFG3L2*, *AIMP1*, *ALDH18A1*, *ALDH3A2*, *ALS2*, *ANG*, *ANO10*, *ANO3*, *AP4B1*, *AP4E1*, *AP4M1*, *AP4S1*, *AP5Z1*, *APOE*, *APP*, *APTX*, *ARSA*, *ASPA*, *ATCAY*, *ATL1*, *ATM*, *ATP13A2*, *ATP1A2*, *ATP1A3*, *ATP7B*, *ATP8A2*, *ATXN2*, *B4GALNT1*, *BOLA3*, *BSCL2*, *CA2*, *CA8*, *CACNA1A*, *CACNA1G*, *CACNB4*, *CAMTA1*, *CCDC88C*, *CHMP2B*, *CIZ1*, *CLCN2*, *CLN3*, *CLN5*, *CLN6*, *CLN8*, *COL4A1*, *COL4A2*, *COL6A3*, *COQ8A*, *COX10*, *COX15*, *COX6B1*, *CP*, *CSF1R*, *CTC1*, *CTSA*, *CYP27A1*, *CYP2U1*, *CYP7B1*, *DARS1*, *DARS2*, *DCAF17*, *DCTN1*, *DDHD1*, *DDHD2*, *DNAJC6*, *DNMT1*, *DRD3*, *DSTYK*, *EARS2*, *EIF2B1*, *EIF2B2*, *EIF2B3*, *EIF2B4*, *EIF2B5*, *EIF4G1*, *ELOVL4*, *ELOVL5*, *EMC1*, *EPRS1*, *ERCC6*, *ERCC8*, *ERLIN2*, *FA2H*, *FAM126A*, *FARS2*, *FBXO7*, *FGF14*, *FIG4*, *FKRP*, *FKTN*, *FLVCR1*, *FOLR1*, *FOXC1*, *FOXRED1*, *FUCA1*, *FUS*, *FXN*, *GALC*, *GAN*, *GBA*, *GBE1*, *GCDH*, *GCH1*, *GFAP*, *GFM1*, *GIGYF2*, *GJC2*, *GLA*, *GLB1*, *GLRX5*, *GOSR2*, *GRID2*, *GRM1*, *GRN*, *HEPACAM*, *HEXA*, *HEXB*, *HSD17B4*, *HTRA1*, *HTRA2*, *IDS*, *IFIH1*, *ITM2B*, *ITPR1*, *KARS1*, *KCNA1*, *KCNC3*, *KCND3*, *KCNJ10*, *KCNMA1*, *KCNT1*, *KCTD7*, *KIF1A*, *KIF5A*, *L1CAM*, *L2HGDH*, *LAMA2*, *LARGE1*, *LMNB1*, *LRPPRC*, *LRRK2*, *MAPT*, *MARS2*, *MATR3*, *MCOLN1*, *MFSD8*, *MLC1*, *MPV17*, *MRE11*, *MTFMT*, *MTHFS*, *MTTP*, *NARS2*, *NDUFS1*, *NDUFV1*, *NFU1*, *NIPA1*, *NKX2-1*, *NOTCH3*, *NOTCH2NLC*, *NPC1*, *NPC2*, *NUBPL*, *OCLN*, *OPTN*, *PANK2*, *PARK7*, *PC*, *PDGFB*, *PDGFRB*, *PDYN*, *PEX1*, *PEX10*, *PEX11B*, *PEX12*, *PEX13*, *PEX14*, *PEX16*, *PEX19*, *PEX2*, *PEX26*, *PEX3*, *PEX5*, *PEX6*, *PEX7*, *PFN1*, *PHGDH*, *PHYH*, *PINK1*, *PLA2G6*, *PLP1*, *PNKD*, *PNKP*, *PNPLA6*, *POLG*, *POLR1C*, *POLR3A*, *POLR3B*, *POMGNT1*, *POMT1*, *POMT2*, *PPT1*, *PRICKLE1*, *PRKCG*, *PRKN*, *PRNP*, *PRRT2*, *PSAP*, *PSAT1*, *PSEN1*, *PSEN2*, *RARS2*, *REEP1*, *RNASEH2A*, *RNASEH2B*, *RNASEH2C*, *RNASET2*, *RNF170*, *RTN2*, *SACS*, *SAMHD1*, *SCN1A*, *SCN2A*, *SCN8A*, *SCP2*, *SDHA*, *SDHAF1*, *SETX*, *SGCE*, *SIGMAR1*, *SIL1*, *SLC16A2*, *SLC17A5*, *SLC19A3*, *SLC1A3*, *SLC20A2*, *SLC25A12*, *SLC2A1*, *SLC30A10*, *SLC33A1*, *SLC6A3*, *SNCA*, *SOD1*, *SOX10*, *SPART*, *SPAST*, *SPG11*, *SPG21*, *SPG7*, *SPR*, *SPTAN1*, *SPTBN2*, *SQSTM1*, *SUMF1*, *SURF1*, *SYNE1*, *TACO1*, *TARDBP*, *TBCD*, *TBCK*, *TBK1*, *TBP*, *TDP1*, *TECPR2*, *TFG*, *TGFB1*, *TGM6*, *TH*, *THAP1*, *TOR1A*, *TPP1*, TRAPPC9, TYMP, TYROBP, UBQLN2, *UCHL1*, *VAPB*, *VCP*, *VLDLR*, *VPS13A*, *VPS35*, *WASHC5*, *WDR45*, *WDR45B*, *WDR81*, *WFS1*, *WWOX*, *XK*, *XRCC1*, *ZFYVE26*) associated with neurodegenerative diseases. Afterwards Human Phenotype Ontology was used for phenotypic filters, and Online Mendelian Inheritance in Man (OMIM, https://www.omim.org/) was used for gene sets. The Human Genome Mutation Database (HGMD, http://www.hgmd.cf.ac.uk/ac/index.php), Franklin (https://franklin.genoox.com/clinical-db/home), VarSome (https://varsome.com/) and novel variants in the databases were checked. The pathogenicity score of new variants was interpreted using the in silico variant prediction programme Mutation Taster, Combined Annotation Dependent Depletion.
